# Primary site as a novel prognostic factor for cardiovascular mortality post-radiotherapy in limited-stage small cell lung cancer: A large population-based study

**DOI:** 10.3389/fcvm.2022.922811

**Published:** 2022-08-12

**Authors:** Yuwei Zhao, Fen Qin, Qingqi Ji, Wuyan Xia, Ben He

**Affiliations:** ^1^Department of Cardiology, Shanghai Chest Hospital, Shanghai Jiao Tong University, Shanghai, China; ^2^Department of Cardiology, The First Affiliated Hospital of Zhengzhou University, Zhengzhou, China; ^3^Department of Radiation Oncology, Shanghai Chest Hospital, Shanghai Jiao Tong University, Shanghai, China; ^4^Clinical Research Center, Shanghai Jiao Tong University School of Medicine, Shanghai, China

**Keywords:** small cell lung cancer, radiotherapy, tumor primary site, cardiovascular mortality, SEER

## Abstract

**Background:**

The effect of primary site on cardiovascular mortality (CVM) post-radiotherapy (RT) in patients with limited-stage small cell lung cancer (LS-SCLC) remains unclear.

**Methods:**

We screened the Surveillance, Epidemiology, and End Results (SEER) database between 1988 and 2013. We used cumulative incidence function (CIF) curves to compare CVM incidences, and performed Cox proportional hazards and Fine-Gray competing risk analyses to identify independent risk factors of CVM. Propensity score matching (PSM) analysis was conducted.

**Results:**

Among enrolled 4,824 patients (median age 57 years old, 49.2% were male), CVM accounts for 10.0% of all deaths after 5 years since cancer diagnosis. Hazard ratios (HRs) for CVM were 1.97 (95% CI: 1.23–3.16, *P* = 0.005) for main bronchus (MB) patients, 1.65 (95% CI: 1.04–2.63, *P* = 0.034) for lower lobe (LL) patients and 1.01 (95% CI: 0.40–2.59, *P* = 0.977) for middle lobe (ML) patients compared to upper lobe (UL) patients. CIF curves showed that the cumulative CVM incidence was greater in the re-categorized MB/LL group compared to UL/ML group both before PSM (*P* = 0.005) and after PSM (*P* = 0.012). Multivariate regression models indicated that MB/LL was independently associated with an increased CVM risk, before PSM (HR_Cox_: 1.79, 95% CI: 1.23–2.61, *P* = 0.002; HR_Fine−Gray_: 1.71, 95% CI: 1.18–2.48, *P* = 0.005) and after PSM (HR_Cox_: 1.88, 95% CI: 1.20–2.95, *P* = 0.006; HR_Fine−Gray_: 1.79, 95% CI: 1.15–2.79, *P* = 0.010).

**Conclusions:**

MB/LL as the primary site is independently associated with an increased CVM risk post-RT in patients with LS-SCLC.

## Introduction

Radiotherapy (RT) is frequently used as an essential adjuvant to chemotherapy or surgery in thoracic malignancies. RT has been shown to improve cancer-specific survival; however, it has been implicated in pulmonary and cardiac complications because of reported acute and chronic radiation-induced injuries to healthy tissues in the radiation field (1–4). Some reports have focused on cardiovascular toxicities post-thoracic RT in long-term cancer survivors, including those with breast cancer and Hodgkin lymphoma (3, 5–7).

Lung cancer is a major malignancy that accounts for the highest morbidity and mortality rates worldwide (8). Adverse effects of RT on the cardiovascular system in patients with lung cancer have recently attracted wider attention and have gained increasing interest in the field of cardio-oncology. Previous studies have shown that RT could increase the incidence of cardiovascular complications in patients with non-small cell lung cancer (NSCLC) (9–14). However, investigations on RT-related cardiovascular sequelae in patients with limited-stage small cell lung cancer (LS-SCLC) remain scarce. This may be partially because, historically, LS-SCLC was considered to have an unfavorable median overall survival (OS) of approximately 1 year before the 1990s (15). Nevertheless, survival rates for patients with LS-SCLC have gradually improved due to wide-spread application of early chest CT screening in high-risk populations, advanced modern RT techniques, more accurate staging paradigms, and recent promising treatment strategies (16, 17). Thoracic RT combined with chemotherapy (CTX) is considered the first-line standard therapy for LS-SCLC (16, 17). However, more extensive studies are needed to evaluate RT-related cardiovascular toxicities in patients with LS-SCLC.

The primary site as a conventional clinical characteristic affecting a lung cancer treatment strategy has currently been recognized as an important prognostic factor for OS and tumor-specific prognosis (18–20). With a disparity in the distance between tumor location and heart/great vessels, potential RT-induced cardiovascular injury may be further distinctive risk (21); however, few relevant reports are available.

This study aimed to identify significant prognostic factors concerning CVM post-RT for patients with LS-SCLC, and to explore the effect of different primary site-based RTs on CVM in a large population of patients with LS-SCLC using data from the Surveillance, Epidemiology, and End Results (SEER) database.

## Materials and methods

### Patients and data sources

**Figure 1:**
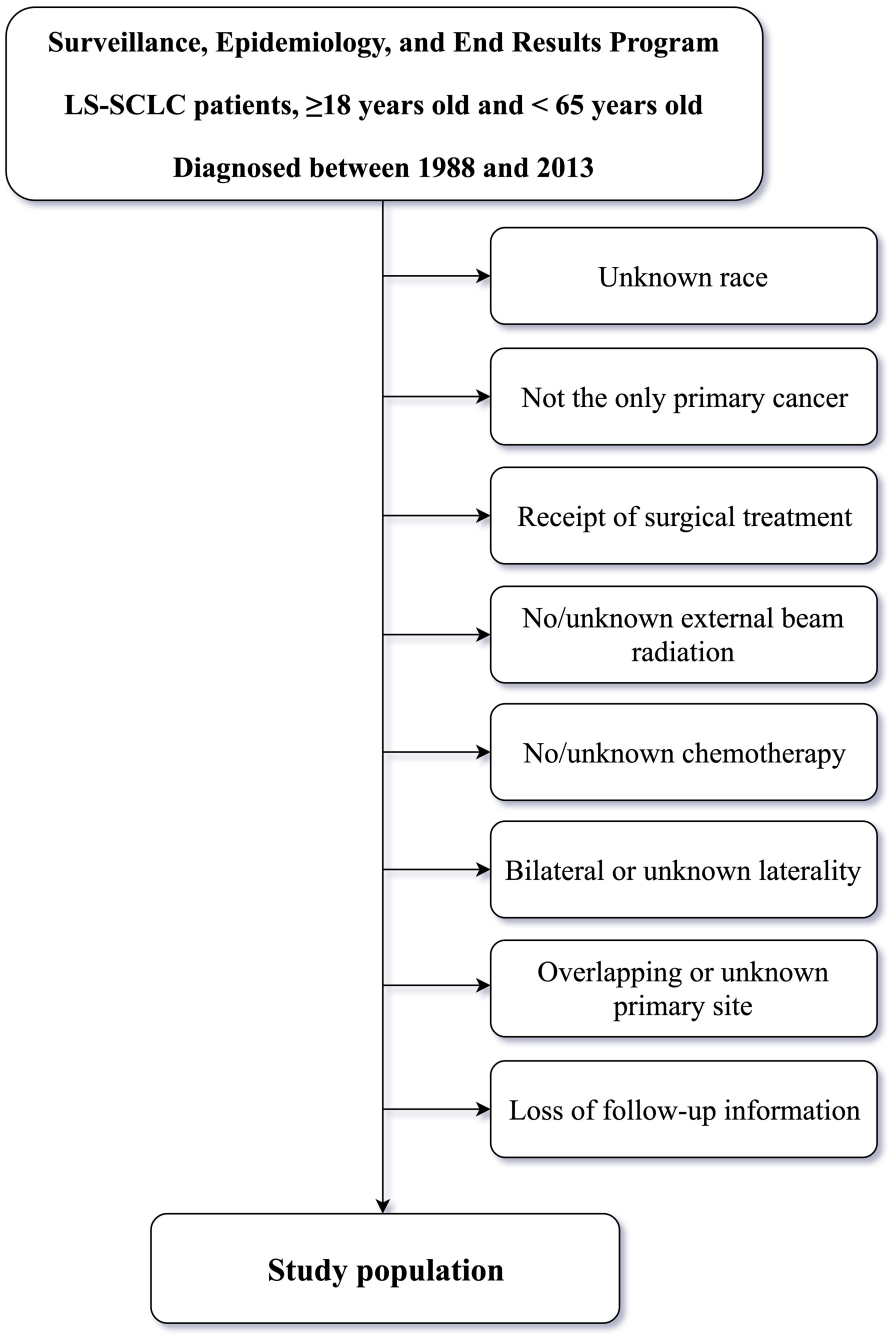
The flow chart of inclusion and exclusion criteria for the study population.

The SEER database [SEER 18 Regs Custom Data (with additional treatment fields), November 2018 Sub] was queried using SEER^*^Stat software (version 8.3.6). Inclusion criteria for patients were as follows: patients aged ≥18 and <65 years and diagnosed with LS-SCLC between 1988 and 2013; patients treated with external beam RT, and; patients with only one primary tumor, a positive histology, available clinical information, active follow-up, and complete dates. Older adult patients with more confounding factors such as coronary heart disease (CHD), hyperlipidemia, hypertension, and diabetes mellitus (DM) were not enrolled for the purpose of alleviating, at least partially, the effects of confounders on CVM in patients post-RT (1, 2, 14), and because younger patients are reported to be more vulnerable to radiation-induced cardiovascular injury (22). The specific time period of 1988–2013 was selected because American Joint Committee on Cancer (AJCC) staging for SCLC in the SEER database started in 1988, whereas 2013 was the final year for analysis in which adequate follow-up to assess post-treatment CVM was possible. Exclusion criteria included: unknown race, not the only primary cancer, receipt of surgical treatment, no/unknown external beam radiation, no/unknown chemotherapy, bilateral or unknown laterality, overlapping or unknown primary site or loss of follow-up information. Inclusion and exclusion criteria for the study population is outlined in Figure 1.

This study was conducted in accordance with the Declaration of Helsinki (as revised in 2013) and complied with the requirements of the Institutional Review Board of Shanghai Chest Hospital, Shanghai Jiao Tong University. The authors have gotten the access to and approval from the SEER database (accession and approval number: 13236-November 2019). The need for informed consent has been waived due to the retrospective nature of the study and because SEER database is a public anonymized database.

### Definition of LS-SCLC

LS-SCLC was defined as AJCC stage I-III malignancies with primary sites in the lung or bronchus [International Classification of Diseased for Oncology-3 (ICD-O-3)/WHO 2008: Lung and Bronchus]. Histological types were as follows: ICD-O-3 codes: 8002, 8041-8045. Primary sites were as follows: main bronchus (MB) (C34.0), upper lobe (UL) (C34.1), middle lobe (ML) (C34.2), and lower lobe (LL) (C34.3).

### Research variables

Demographics and clinicopathologic data, such as age, sex, race, marriage, year of diagnosis, AJCC stage, laterality and primary site were collected. CVM was defined as death due to cardiovascular diseases using the following ICD-10 codes: I00-I52 and I70-I79, including conditions such as diseases of the heart, hypertension without heart disease, atherosclerosis, aortic aneurysm and dissection, and other diseases of the arteries, arterioles, and capillaries. Non-cardiovascular mortality (NCVM) was defined as death due to other causes, excluding CVM.

### Statistical analysis

Statistical analysis was performed using either R (version 3.6.0, R Foundation for Statistical Computing, Vienna, Austria) or Stata (version 15.0, College Station, Texas, USA) software. All statistical tests were two-sided, and the significance level was set at 0.05. As the only continuous variable, age was expressed as median [with inter-quartile range (IQR)] for non-normally distributed data and compared using a Kruskal-Wallis test between the groups. Categorical variables were expressed as numbers (percentages) and then compared using a chi-square test.

We generated cumulative incidence function (CIF) curves using univariate Fine-Gray competing risk regression models to compare the cumulative incidences of CVM or NCVM between the groups. Univariate and multivariate Cox proportional hazards regression models were applied to identify factors associated with CVM or NCVM risk. Based on results obtained from multivariate Cox proportional hazards regression models, the UL and the ML as primary sites were re-categorized into a UL/ML group, and the MB and the LL were combined to form a MB/LL group. Accounting for mortality from other causes, univariate and multivariate Fine-Gray competing risk regression models (23) were used to validate factors associated with CVM risk and obtain more accurate results. The propensity score matching (PSM) method (24, 25) was used to balance the baseline bias between the UL/ML and MB/LL groups. A greedy matching algorithm was used for PSM and the caliper was set at 0.02.

## Results

### Patient demographics and clinical characteristics

A flowchart indicating inclusion and exclusion for the study population is outlined in Figure 1. We enrolled 4,824 patients with LS-SCLC {median age, 57 [interquartile range (IQR), 52–61] years; males, 49.2%}, of whom 2,487 (51.6%) were ≤57 years old, 2,373 (49.2%) were male, and 84.4% were of White ethnicity. There were 1,957 (40.6%) and 2,717 (56.3%) unmarried and married patients, respectively. In addition, 2,118 (43.9%) patients had been diagnosed with LS-SCLC in the 1988–2003 period, and 2,706 (56.1%) patients in the 2004–2013 period. In terms of AJCC stage, 15.0% and 85.0% of patients were classified in Stage I-II and Stage III, respectively. In terms of laterality, 1,965 (40.7%) patients had left-sided tumors and 2,859 (59.3%) had right-sided tumors. In terms of primary site tumor location, 2,890 (59.9%) patients had primary site tumors in the UL, 247 (5.1%) patients had primary site tumors in the ML, 748 (15.5%) patients had primary site tumors in the MB, and 939 (19.5%) patients had primary site tumors in the LL.

**Figure 2:**
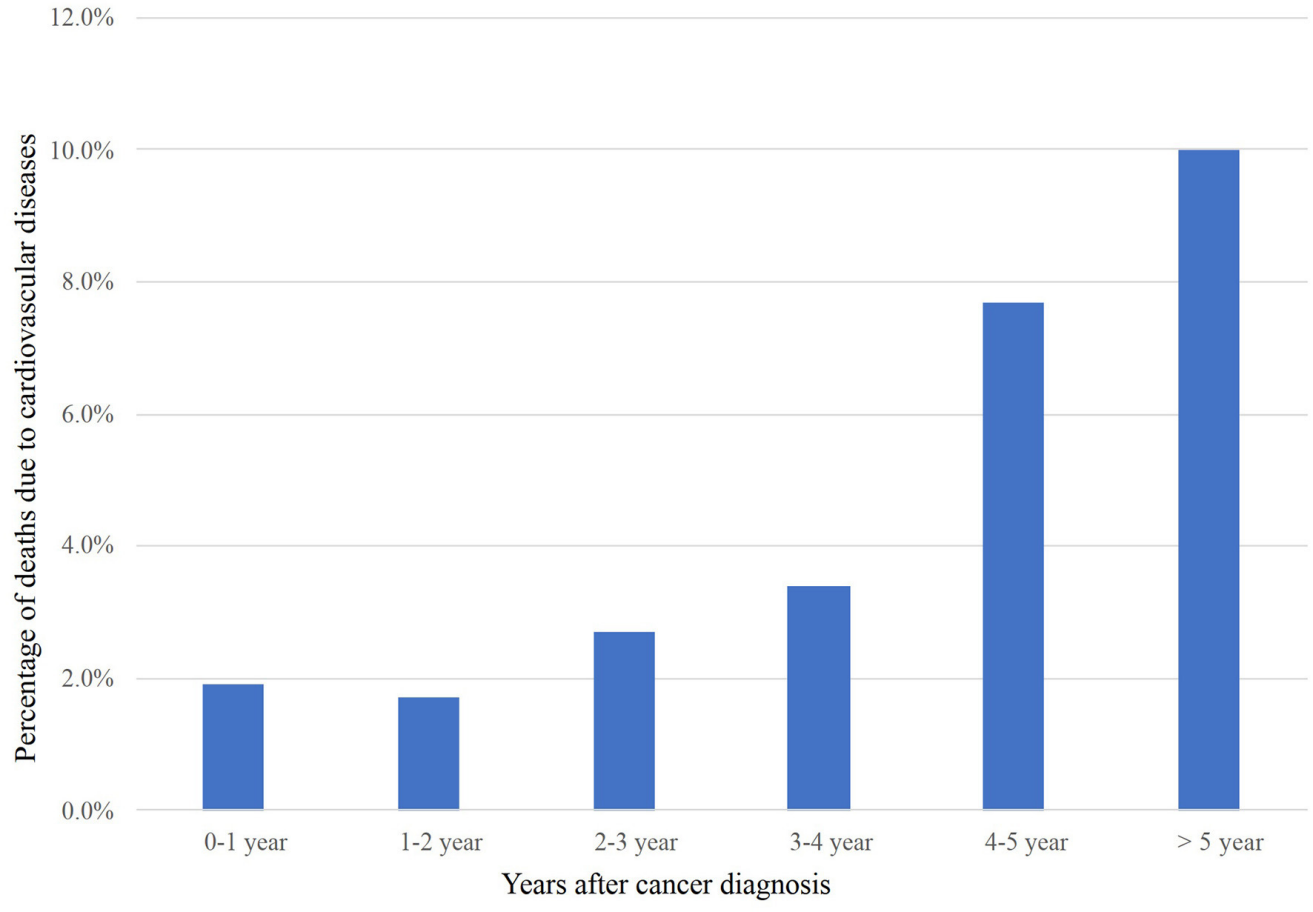
CVM post-radiotherapy as a proportion of all deaths within a given time period after LS-SCLC diagnosis. CVM, cardiovascular mortality; LS-SCLC, limited-stage small cell lung cancer.

The overall incidence of CVM and NCVM at the follow-up endpoint (November 2018) was 2.3 and 87.3%, respectively. Baseline clinical and prognostic characteristics concerning the study population are shown in Table 1. The percentage of deaths due to cardiovascular diseases following diagnosis was tabulated (Figure 2). Within the first year of diagnosis, 1.9% of all deaths were CVM-related and this percentage increased from 1.7% in year two to 2.7% in year three, to 3.4% in year four, to 7.7% in year five, and to 10.0% after 5 years, showing an increasing trend for percentage of deaths due to CVM along with patients' survival time.

**Table 1:** The baseline clinical and prognostic characteristics of total study population.

**Variables**	**Number**	**%**
Total	4,824	100.0%
**Primary site**		
Upper lobe	2,890	59.9%
Middle lobe	247	5.1%
Main bronchus	748	15.5%
Lower lobe	939	19.5%
**Age, years**		
≤57	2,487	51.6%
>57	2,337	48.4%
**Sex**		
Male	2,373	49.2%
Female	2,451	50.8%
**Race**		
White	4,071	84.4%
Black	538	11.2%
Other	215	4.5%
**Marriage**		
Unmarried	1,957	40.6%
Married	2,717	56.3%
Unknown	150	3.1%
**Year of diagnosis**		
1988–2003	2,118	43.9%
2004–2013	2,706	56.1%
**AJCC stage**		
I-II	724	15.0%
III	4,100	85.0%
**Laterality**		
Left	1,965	40.7%
Right	2,859	59.3%
**Prognosis**		
CVM	113	2.3%
NCVM	4,212	87.3%
Alive	499	10.3%

AJCC, American Joint Committee on Cancer; CVM, cardiovascular mortality; NCVM, non-cardiovascular mortality. Percentages might not add up to 100% because of rounding.

### Analysis of CVM based on different variables

**Figure 3:**
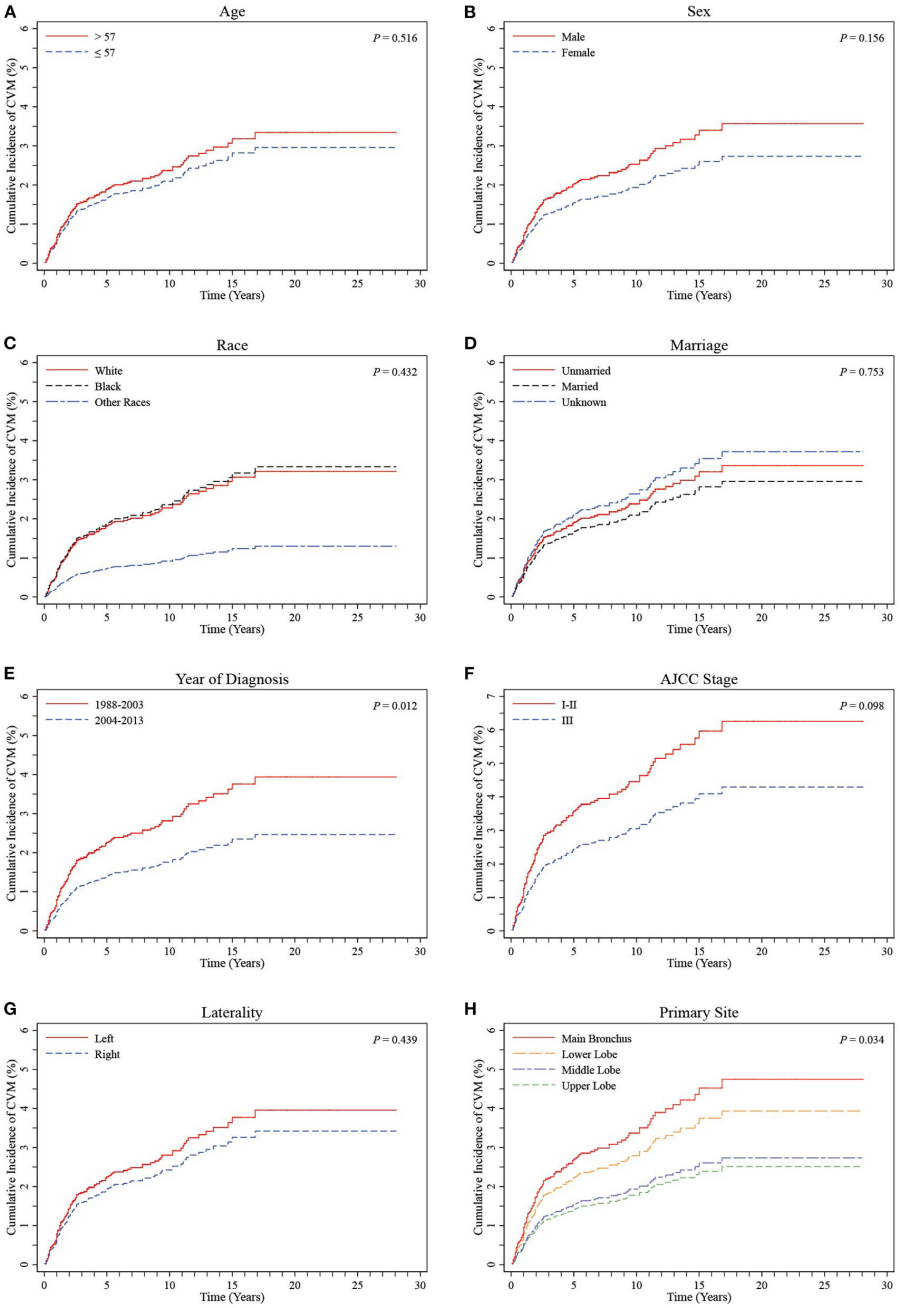
CIF curves of CVM by different variables in LS-SCLC patients. **(A)** Age; **(B)** Sex; **(C)** Race; **(D)** Marriage; **(E)** Year of diagnosis; **(F)** AJCC stage; **(G)** Laterality; **(H)** Primary site (stratified into UL, ML, MB and LL groups). CIF: cumulative incidence function; CVM: cardiovascular mortality; AJCC, American Joint Committee on Cancer; LS-SCLC, limited-stage small cell lung cancer.

Primary sites were initially divided into UL, ML, MB, and LL groups. CIF curves showed no significant differences in cumulative incidences of CVM between groups according to age, sex, ethnicity, marital status, AJCC stage, or laterality (all *P* > 0.05, Figures 3A–D,F,G). A comparison between time periods for diagnosis showed a significantly higher cumulative incidence of CVM in the 1988-2003 period relative to the 2004–2013 period (*P* = 0.012, Figure 3E). Additionally, there was a significant difference between the four groups in terms of the primary sites (*P* = 0.034, Figure 3H).

Multivariate Cox proportional hazards regression models showed independent predictors of CVM risk in patients with LS-SCLC, including sex [female vs. male: hazard ratio (HR) 0.59, 95% confidence interval (CI) 0.40–0.87; *P* = 0.007], time period for diagnosis (2004–2013 vs. 1988–2003, HR 0.64, 95% CI 0.43–0.95; *P* = 0.028), and primary site (ML *vs*. UL, HR 1.01, 95% CI 0.40–2.59, *P* = 0.977; MB vs. UL, HR 1.97, 95% CI 1.23–3.16, *P* = 0.005, and; LL vs. UL, HR 1.65, 95% CI 1.04–2.63, *P* = 0.034). A summary of the results of Cox proportional hazards regression models used to predict CVM risk are listed in Table 2.

**Table 2:** Cox proportional hazards regression models for predictors of CVM.

**Variables**	**Group**	**Cox proportional hazards (Univariate)**		**Cox proportional hazards (Multivariate)**	
		**HR (95% CI)**	***P*-value**	**HR (95% CI)**	***P*-value**
Primary site	Upper lobe	Reference		Reference	
	Middle lobe	0.97 (0.39–2.44)	0.957	1.01 (0.40–2.59)	0.977
	Main bronchus	1.91 (1.20–3.06)	0.007	1.97 (1.23–3.16)	0.005
	Lower lobe	1.65 (1.04–2.63)	0.033	1.65 (1.04–2.63)	0.034
Age, years	≤57	Reference		Reference	
	>57	1.41 (0.97–2.05)	0.071	1.45 (1.00–2.12)	0.052
Sex	Male	Reference		Reference	
	Female	0.64 (0.44–0.94)	0.021	0.59 (0.40–0.87)	0.007
Race	White	Reference		Reference	
	Black	1.15 (0.65–2.06)	0.631	1.07 (0.60–1.93)	0.813
	Other	0.41 (0.10–1.67)	0.213	0.37 (0.09–1.49)	0.161
Marriage	Unmarried	Reference		Reference	
	Married	0.75 (0.51–1.11)	0.150	0.70 (0.47–1.04)	0.075
	Unknown	1.01 (0.36–2.81)	0.982	1.03 (0.37–2.87)	0.953
Year of diagnosis	1988–2003	Reference		Reference	
	2004–2013	0.63 (0.42–0.92)	0.019	0.64 (0.43–0.95)	0.028
AJCC stage	I–II	Reference		Reference	
	III	0.94 (0.59–1.50)	0.808	1.03 (0.64–1.64)	0.913
Laterality	Left	Reference		Reference	
	Right	0.83 (0.57–1.20)	0.316	0.82 (0.56–1.21)	0.327

CVM, cardiovascular mortality; HR, hazard ratio; CI, confidence interval; AJCC, American Joint Committee on Cancer.

### Analysis of CVM based on primary site stratification across UL/ML and MB/LL groups before and after PSM

**Figure 4:**
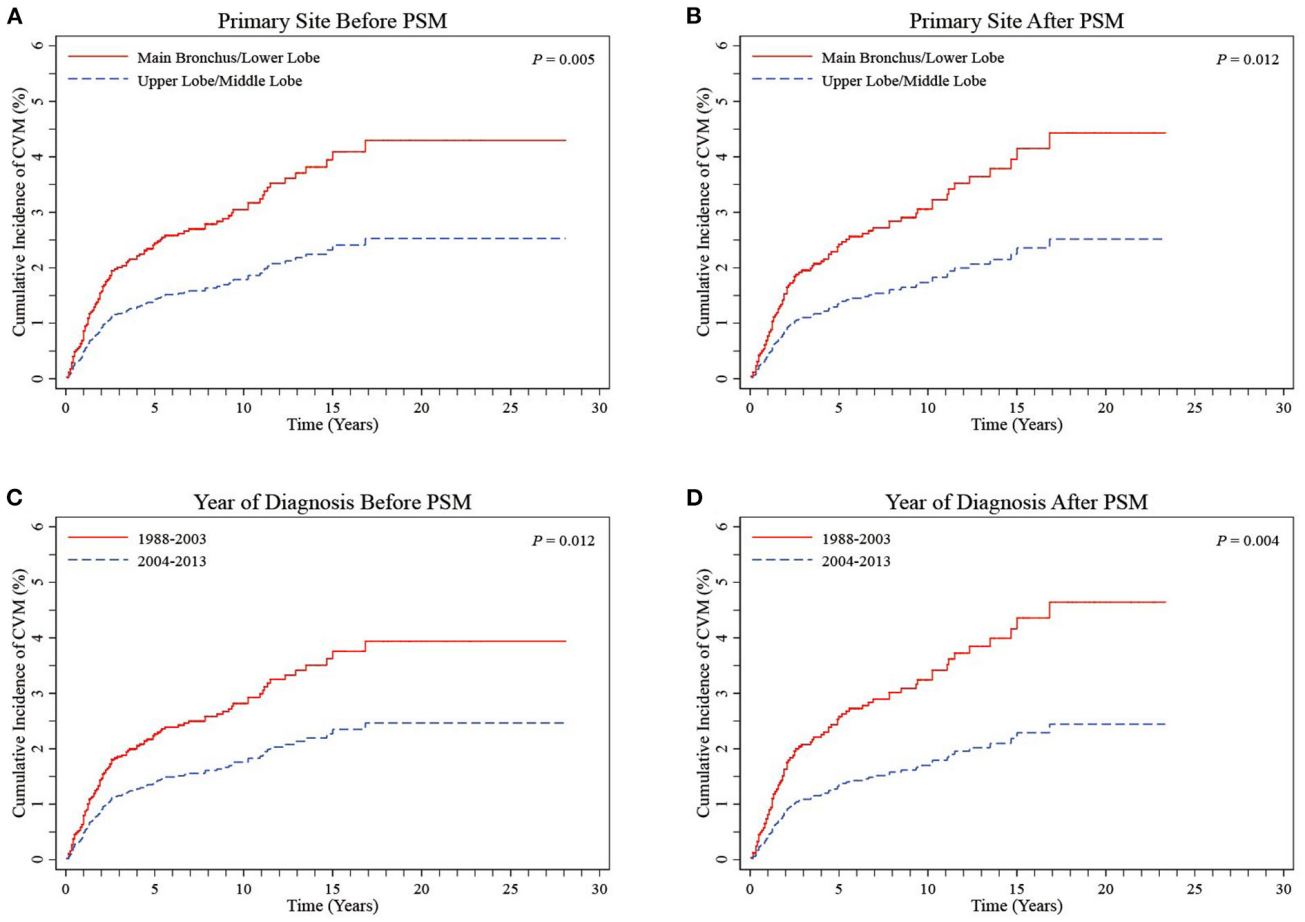
CIF curves of CVM stratified into UL/ML and MB/LL groups by primary site before (**A)** and after **(B)** PSM and stratified into 1988–2003 and 2004–2013 groups by year of diagnosis before **(C)** and after **(D)** PSM in LS-SCLC patients. CIF, cumulative incidence function; CVM, cardiovascular mortality; UL/ML, upper lobe/middle lobe; MB/LL, main bronchus/lower lobe; PSM, propensity score matching; LS-SCLC, limited-stage small cell lung cancer.

Based on the results obtained from multivariate Cox proportional hazards regression models, patients with UL and ML primary site tumors were grouped together into a UL/ML group, and patients with MB and LL primary site tumors were combined to form an MB/LL group. The proportion of patients with left-sided primary site tumors was significantly higher in the MB/LL group than in the UL/ML group before PSM (45.1 vs. 38.4%, *P* < 0.001). To prevent baseline bias, 1,687 patients in the UL/ML group were matched 1:1 with those from the MB/LL group using the PSM method, which showed a good match in terms of demographic and clinicopathologic characteristics (Table 3). We found a higher CVM incidence at the end of the follow-up (November 2018) in patients in the MB/LL group compared to those in the UL/ML group. We observed a before PSM CVM incidence of 3.2% and 1.9% (*P* = 0.005) in the MB/LL and UL/ML groups, respectively, and 3.2 and 1.8% (*P* = 0.011) after PSM, respectively (Table 3). CIF curves showed that the cumulative CVM incidence was significantly higher in the MB/LL group than in the UL/ML group before PSM (*P* = 0.005, Figure 4A) and after PSM (*P* = 0.012, Figure 4B).

**Table 3:** The baseline clinical and prognostic characteristics of LS-SCLC patients stratified into UL/ML and MB/LL groups by primary site before and after PSM.

**Variables**	**Before PSM**			**After PSM**		
	**UL/ML**	**MB/LL**	***P*-value**	**UL/ML**	**MB/LL**	***P*-value**
	**(*n* = 3,137)**	**(*n* = 1,687)**		**(*n* = 1,687)**	**(*n* = 1,687)**	
Age, years			0.915			0.470
≤ 57, no. (%)	1,615 (51.5%)	872 (51.7%)		850 (50.4%)	872 (51.7%)	
>57, no. (%)	1,522 (48.5%)	815 (48.3%)		837 (49.6%)	815 (48.3%)	
Sex			0.158			0.148
Male, no. (%)	1,567 (50.0%)	806 (47.8%)		849 (50.3%)	806 (47.8%)	
Female, no. (%)	1,570 (50.0%)	881 (52.2%)		838 (49.7%)	881 (52.2%)	
Race			0.696			0.704
White, no. (%)	2,652 (84.5%)	1,419 (84.1%)		1,430 (84.8%)	1,419 (84.1%)	
Black, no. (%)	351 (11.2%)	187 (11.1%)		186 (11.0%)	187 (11.1%)	
Other, no. (%)	134 (4.3%)	81 (4.8%)		71 (4.2%)	81 (4.8%)	
Marriage			0.892			0.994
Unmarried, no. (%)	1,275 (40.6%)	682 (40.4%)		683 (40.5%)	682 (40.4%)	
Married, no. (%)	1,762 (56.2%)	955 (56.6%)		953 (56.5%)	955 (56.6%)	
Unknown, no. (%)	100 (3.2%)	50 (3.0%)		51 (3.0%)	50 (3.0%)	
Year of diagnosis			0.401			0.603
1988–2003, no. (%)	1,363 (43.4%)	755 (44.8%)		739 (43.8%)	755 (44.8%)	
2004–2013, no. (%)	1,774 (56.6%)	932 (55.2%)		948 (56.2%)	932 (55.2%)	
AJCC stage			0.537			0.206
I–II, no. (%)	463 (14.8%)	261 (15.5%)		234 (13.9%)	261 (15.5%)	
III, no. (%)	2,674 (85.2%)	1,426 (84.5%)		1,453 (86.1%)	1,426 (84.5%)	
Laterality			<0.001			1.000
Left, no. (%)	1,205 (38.4%)	760 (45.1%)		760 (45.1%)	760 (45.1%)	
Right, no. (%)	1,932 (61.6%)	927 (54.9%)		927 (54.9%)	927 (54.9%)	
Prognosis						
CVM	59 (1.9%)	54 (3.2%)	0.005	30 (1.8%)	54 (3.2%)	0.011
NCVM	2,752 (87.7%)	1,460 (86.5%)	0.239	1,472 (87.3%)	1,460 (86.5%)	0.540
Alive	326 (10.4%)	173 (10.3%)	0.881	185 (11.0%)	173 (10.3%)	0.273

LS-SCLC, limited-stage small cell lung cancer; UL/ML, upper lobe/middle lobe; MB/LL, main bronchus/lower lobe; PSM, propensity score matching; AJCC, American Joint Committee on Cancer; CVM, cardiovascular mortality; NCVM, non-cardiovascular mortality. Percentages might not add up to 100% because of rounding.

Regression analyses showed that MB/LL primary site tumors were independently associated with an increased CVM risk compared with UL/ML primary site tumors in patients with LS-SCLC before and after PSM (Tables 4, 5). Specifically, multivariate Cox models showed an HR of 1.79 (95% CI 1.23–2.61, *P* = 0.002), whereas multivariate Fine-Gray models indicated an HR of 1.71 (95% CI 1.18–2.48, *P* = 0.005) before PSM in patients with LS-SCLC in the MB/LL group compared with those in the UL/ML group (Table 4). After PSM, an HR of 1.88 (95% CI 1.20–2.95, *P* = 0.006) and an HR of 1.79 (95% CI 1.15–2.79, *P* = 0.010) were recorded for multivariate Cox proportional hazards regression and Fine-Gray models, respectively, for patients with LS-SCLC in the MB/LL group compared with those in the UL/ML group (Table 4).

**Table 4:** Univariate Cox proportional hazards and Fine-Gray competing risk regression models for predictors of CVM before and after PSM.

**Variables**	**Group**	**Before PSM**				**After PSM**			
		**Cox proportional hazards (Univariate)**		**Fine-gray competing risk (Univariate)**		**Cox proportional hazards (Univariate)**		**Fine-gray competing risk (Univariate)**	
		**HR (95% CI)**	***P-*value**	**HR (95% CI)**	***P-*value**	**HR (95% CI)**	***P-*value**	**HR (95% CI)**	***P-*value**
Primary site	UL/ML	Reference		Reference		Reference		Reference	
	MB/LL	1.78 (1.22–2.58)	0.003	1.71 (1.18–2.49)	0.005	1.86 (1.19–2.91)	0.007	1.78 (1.14–2.78)	0.012
Age, years	≤57	Reference		Reference		Reference		Reference	
	>57	1.41 (0.97–2.05)	0.071	1.13 (0.78–1.64)	0.516	1.58 (1.02–2.44)	0.039	1.25 (0.81–1.92)	0.310
Sex	Male	Reference		Reference		Reference		Reference	
	Female	0.64 (0.44–0.94)	0.021	0.76 (0.52–1.11)	0.156	0.60 (0.39–0.94)	0.024	0.73 (0.48–1.13)	0.161
Race	White	Reference		Reference		Reference		Reference	
	Black	1.15 (0.65–2.06)	0.631	1.04 (0.58–1.85)	0.904	1.53 (0.83–2.83)	0.173	1.35 (0.73–2.50)	0.336
	Other	0.41 (0.10–1.67)	0.213	0.40 (0.10–1.62)	0.199	0.56 (0.14–2.31)	0.426	0.56 (0.14–2.26)	0.412
Marriage	Unmarried	Reference		Reference		Reference		Reference	
	Married	0.75 (0.51–1.11)	0.150	0.88 (0.60–1.28)	0.503	0.73 (0.47–1.13)	0.156	0.87 (0.56–1.35)	0.527
	Unknown	1.01 (0.36–2.81)	0.982	1.11 (0.40–3.07)	0.844	1.08 (0.33–3.50)	0.900	1.16 (0.36–3.77)	0.800
Year of diagnosis	1988–2003	Reference		Reference		Reference		Reference	
	2004–2013	0.63 (0.42–0.92)	0.019	0.62 (0.43–0.90)	0.012	0.53 (0.34–0.84)	0.007	0.52 (0.34–0.81)	0.004
AJCC stage	I–II	Reference		Reference		Reference		Reference	
	III	0.94 (0.60–1.50)	0.808	0.68 (0.43–1.07)	0.098	0.83 (0.50–1.39)	0.481	0.58 (0.35–0.97)	0.038
Laterality	Left	Reference		Reference		Reference		Reference	
	Right	0.83 (0.57–1.20)	0.316	0.86 (0.59–1.25)	0.439	0.90 (0.58–1.38)	0.616	0.97 (0.63–1.49)	0.873

CVM, cardiovascular mortality; PSM, propensity score matching; HR, hazard ratio; CI, confidence interval; AJCC, American Joint Committee on Cancer; UL/ML, upper lobe/middle lobe; MB/LL, main bronchus/lower lobe.

**Table 5:** Multivariate Cox proportional hazards and Fine-Gray competing risk regression models for predictors of CVM before and after PSM.

**Variables**	**Group**	**Before PSM**				**After PSM**			
		**Cox proportional hazards (Multivariate)**		**Fine-gray competing risk (Multivariate)**		**Cox proportional hazards (Multivariate)**		**Fine-gray competing risk (Multivariate)**	
		**HR (95% CI)**	***P-*value**	**HR (95% CI)**	***P-*value**	**HR (95% CI)**	***P-*value**	**HR (95% CI)**	***P-*value**
Primary site	UL/ML	Reference		Reference		Reference		Reference	
	MB/LL	1.79 (1.23–2.61)	0.002	1.71 (1.18–2.48)	0.005	1.88 (1.20–2.95)	0.006	1.79 (1.15–2.79)	0.010
Age, years	≤57	Reference		Reference		Reference		Reference	
	>57	1.45 (0.99–2.10)	0.055	1.16 (0.80–1.68)	0.439	1.66 (1.07–2.58)	0.023	1.30 (0.85–2.00)	0.230
Sex	Male	Reference		Reference		Reference		Reference	
	Female	0.60 (0.41–0.87)	0.008	0.73 (0.50–1.07)	0.110	0.57 (0.37–0.89)	0.013	0.71 (0.46–1.10)	0.123
Race	White	Reference		Reference		Reference		Reference	
	Black	1.08 (0.60–1.94)	0.806	1.00 (0.56–1.79)	0.996	1.42 (0.76–2.66)	0.271	1.30 (0.71–2.40)	0.399
	Other	0.37 (0.09–1.49)	0.160	0.37 (0.09–1.49)	0.162	0.49 (0.12–2.00)	0.319	0.50 (0.12–2.06)	0.341
Marriage	Unmarried	Reference		Reference		Reference		Reference	
	Married	0.70 (0.47–1.04)	0.074	0.82 (0.56–1.20)	0.310	0.70 (0.44–1.09)	0.116	0.82 (0.53–1.26)	0.360
	Unknown	1.02 (0.37–2.83)	0.974	1.04 (0.38–2.86)	0.940	1.09 (0.33–3.53)	0.891	1.06 (0.33–3.37)	0.927
Year of diagnosis	1988–2003	Reference		Reference		Reference		Reference	
	2004–2013	0.64 (0.43–0.95)	0.025	0.64 (0.44–0.92)	0.016	0.55 (0.34–0.87)	0.010	0.54 (0.35–0.84)	0.006
AJCC stage	I–II	Reference		Reference		Reference		Reference	
	III	1.04 (0.65–1.66)	0.860	0.71 (0.46–1.12)	0.143	0.94 (0.56–1.58)	0.812	0.61 (0.37–1.01)	0.057
Laterality	Left	Reference		Reference		Reference		Reference	
	Right	0.83 (0.57–1.21)	0.322	0.89 (0.62–1.30)	0.555	0.86 (0.56–1.33)	0.507	0.96 (0.63–1.48)	0.865

CVM, cardiovascular mortality; PSM, propensity score matching; HR, hazard ratio; CI, confidence interval; AJCC, American Joint Committee on Cancer; UL/ML, upper lobe/middle lobe; MB/LL, main bronchus/lower lobe.

CIF curves showed that cumulative CVM incidences were both significantly lower in the 1988–2003 period relative to the 2004–2013 period for diagnosis before PSM (*P* = 0.012, Figure 4C) and after PSM (*P* = 0.004, Figure 4D). Regression analyses, based on Cox proportional hazard regression and Fine-Gray competing risk models, showed that the 2004–2013 period was independently associated with lower CVM risk relative to the 1988–2003 before and after PSM (all *P* > 0.05, Tables 4, 5).

### Analysis of NCVM based on different variables

**Figure 5:**
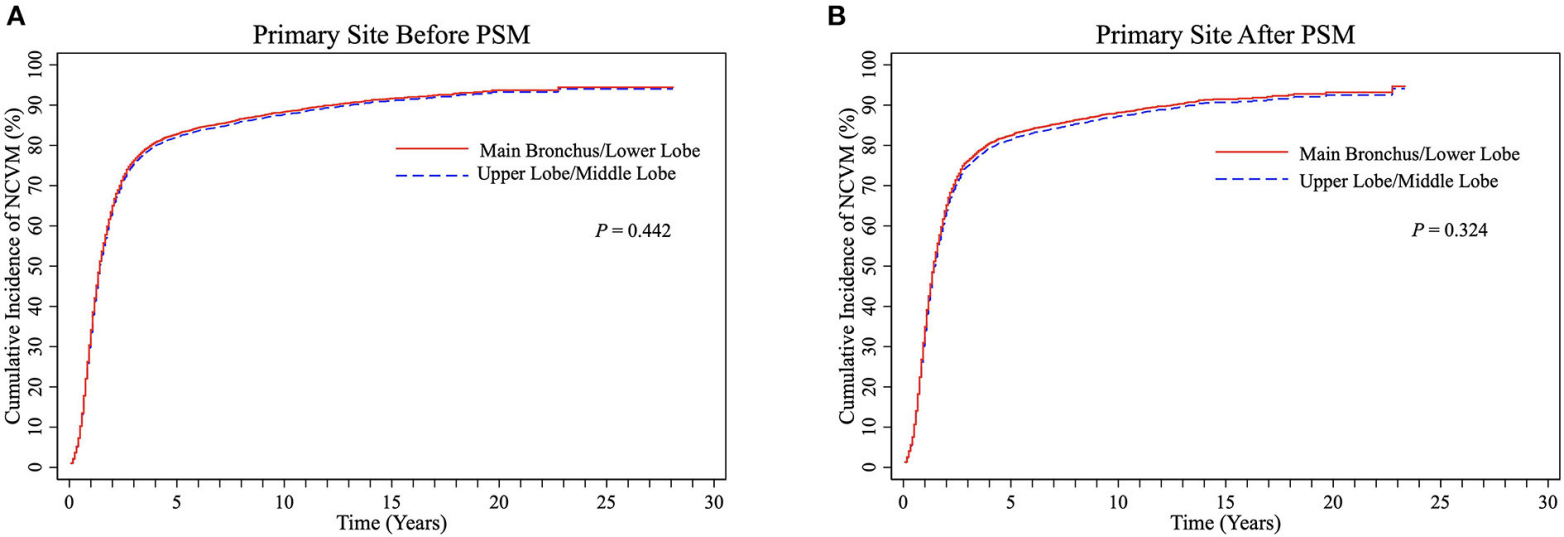
CIF curves of NCVM post-radiotherapy stratified into UL/ML and MB/LL groups by primary site before **(A)** and after **(B)** PSM in LS-SCLC patients. CIF, cumulative incidence function; CVM, cardiovascular mortality; UL/ML, upper lobe/middle lobe; MB/LL, main bronchus/lower lobe; PSM, propensity score matching; LS-SCLC, limited-stage small cell lung cancer.

There were no significant differences in cumulative NCVM incidences between the UL/ML and the LL/MB groups before PSM (*P* = 0.442, Figure 5A) and after PSM (*P* = 0.324, Figure 5B). The univariate and multivariate Fine-Gray competing risk regression analyses showed that primary site was not an independent predictor of NCVM post-RT in patients with LS-SCLC (*P* > 0.05, Supplementary Tables 1, 2).

## Discussion

### Prior works

Studies have shown that RT can increase incidence of cardiovascular complications in lung cancer patients (9–14, 26, 27). For instance, Lally et al. (9) implicated postoperative RT with increased cardiac mortality in NSCLC patients. In the Radiation Therapy Oncology Group (RTOG) 0617 NSCLC trial, heart V5 (volume of heart receiving 5 Gy) and heart V30 were associated with increased risk of cardiac events (CE) as well as inferior survival rates (10). Dess et al. (11) presented a long-term grade 3 CE incidence, exceeding 10%, among a prospective locally advanced NSCLC (LA-NSCLC) cohort. In an analysis of prospective dose-escalation LA-NSCLC trial, Wang et al. (12) demonstrated that the radiation dose delivered to the heart was an independent predictor of CE. In addition, results of a SEER database analysis (13) among 52,624 LA-NSCLC patients receiving thoracic RT, showed that cardiac-specific mortality (CSM) in left-sided patients was significantly higher than that in right-sided patients. A recent study suggested that mean heart dose was a risk factor for major adverse cardiac events and all-cause mortality in a single-institution retrospective cohort study of LA-NSCLC patients (14).

In recent years, research focus has been directed toward long-term RT-related cardiovascular sequelae in patients with SCLC, due to parallels with NSCLC and the rise in life expectancy (16, 17). Ferris et al. (26) performed a data analysis using the SEER database and found that RT was associated with an approximate 10% absolute increase in CE at 5 years in patients with LS-SCLC and multivariate analysis has shown an independent association between RT and CE. A recent SEER database study showed an increased CSM in left vs. right-sided patients with LS-SCLC receiving thoracic RT (27). Currently, no prior study has investigated the effect of primary site on cardiovascular complications especially concerning CVM in patients with LS-SCLC post-RT. Our study has contributed an enhanced understanding to this research field.

### Main findings

This study was the first to report the effects of primary site on CVM post-RT in patients with LS-SCLC. Our results showed that patients in the MB/LL group had a significantly higher cumulative CVM incidence than those in the UL/ML group. MB/LL as the primary site was associated with an increased risk of CVM, and the primary site was a novel prognostic factor for CVM post-RT. This study has many highlights and high reliability of the results. First, we used PSM to balance demographic and clinicopathologic characteristics. These characteristics, especially laterality, have been previously shown to affect occurrence of cardiovascular events in patients with cancer treated with thoracic RT (9, 13, 27). Previous SEER-based analyses involving patients with LA-NSCLC (13) or patients with LS-SCLC (27) receiving thoracic RT showed that CSM in patients with left-sided tumors was significantly higher than that in patients with right-sided tumors. In our study, the percentage of patients with left-sided tumors was significantly greater in the MB/LL patient group than in the UL/ML patient group; therefore, PSM was performed to eliminate possible laterality bias on CVM. Second, rather than using the Kaplan-Meier method, Fine-Gray competing risk regression models (23) that can correctly estimate the probability of an event in the presence of competing events were used in survival analysis to validate the results of Cox proportional hazards regression models. Third, we restricted our analysis to patients aged <65 years. Bias may be present and affect CVM results when comparing patients among all age groups in terms of an unbalanced burden of cardiovascular comorbidities. To address this challenge, we only enrolled patients aged <65 years to help determine any correlation between thoracic RT and CVM risk. We envisaged that this would minimize the effect of underlying cardiovascular risk factors or comorbidities on CVM occurrence. After taking these matters into account, we consider that this study provides a more accurate and reliable evaluation of the effect of thoracic RT on CVM in patients with SCLC.

### Risk factors affecting CVM and potential mechanisms

Thoracic RT has been shown to result in injury to the heart and coronary artery, as well as to other vessels in the radiation field, including the aorta and pulmonary artery, resulting in aortic valve disease, porcelain aorta, and pulmonary artery aneurysm (28–31). The relative anatomical position between a tumor primary site and heart/great vessels might influence the amount of radiation doses received by the heart or great vessels during RT. Anatomically, the LL is closely adjacent to the heart, and has been found to be associated with larger volume variability than the UL during radiation procedures (21). Furthermore, bilateral main bronchi are embedded in the hilum of the lung and are surrounded with several great vessels. Specifically, the right MB passes behind the ascending aorta, the superior vena cava, and the right pulmonary vessels, whereas the left MB passes behind the left pulmonary vessels, and extends across the arch formed by the arch of the aorta and the descending thoracic aorta. This close anatomical relationship makes it more likely for healthy tissues to receive additional radiation exposure, especially heart and great vessel tissues located adjacent to the tumor in patients whose primary site is located in the MB and in the LL, consequently making them more vulnerable to radiation-induced injury. Recent clinical studies have reported that patients with NSCLC with primary sites in the left MB and left LL have lower OS rates (18–21). To date, the specific mechanism to explain this remains unclear, although it may be attributed, at least in part, to increased RT-induced severe adverse cardiovascular events in patients receiving RT with primary sites in the MB and LL (9–14). This explanation accords with our study findings. Two studies have shown that in patients with cancer receiving thoracic RT, left-sided laterality was associated with an increased incidence of cardiovascular complications, due to a shorter distance between the left-sided radiation field and the heart compared with patients with right-sided primary sites (13, 27). This finding provides support for the potential mechanisms involved concerning distinct incidences of CVM in different primary sites in our study.

### Limitations

Although this study provides novel and clinically significant insights into CVM post-RT for patients with non-surgical LS-SCLC, there remain some limitations inherent to any retrospective analysis. First, the SEER database contains limited data concerning pre-existing cardiovascular comorbidities and risk factors. Next, similar to previous RT studies based on data from the SEER database (13, 26), we were unable to assess many important therapeutic parameters, such as total radiation dose, the dose per fraction, the volume of heart/great vessels irradiated, and chemotherapy agents. The validity of reporting RT using SEER data has been questioned. However, one recent study reported a high sensitivity and positive predictive value between RT records and the actual implementation of RT (32). Additionally, given the *post-hoc* nature of this study, limitations in terms of retrospective analyses apply. Nonetheless, MB/LL and UL/ML groups were matched to obviate the potential effect of unbalanced variables on CVM.

## Conclusions

MB/LL as the primary site was found to be associated with an increased risk of CVM post-RT in patients with LS-SCLC. This study presented a propensity score-matched competing risk analysis in a large, population-based, real-world cohort, with which to analyze RT-linked sequelae and to stratify CVM risk during clinical decision-making. Our findings suggested that patients with MB/LL tumors undergoing RT may require better radioprotection not only for the heart, but also for the great vessels. More comprehensive cardiovascular management and closer follow-up are needed for patients with LS-SCLC undergoing RT.

## Data availability statement

The raw data supporting the conclusions of this article will be made available by the authors, without undue reservation.

## Ethics statement

Ethical review and approval was not required for the study on human participants in accordance with the local legislation and institutional requirements. Written informed consent for participation was not required for this study in accordance with the national legislation and the institutional requirements.

## Author contributions

YZ contributed to writing the manuscript, SEER database access, and data acquisition and analysis. YZ, FQ, and BH contributed to the conception, design, and supervision of the study. QJ and WX contributed to data interpretation. All authors contributed to the article and approved the submitted version.

## Funding

This work was supported by the National Natural Science Foundation of China (81830010), the Artificial intelligence project of Xuhui District, Shanghai (No. 2020-011), Clinical Research Plan of SHDC (SHDC2020CR1039B), Shanghai Jiao Tong University School of Medicine clinical research project (dly201512).

## Conflict of interest

The authors declare that the research was conducted in the absence of any commercial or financial relationships that could be construed as a potential conflict of interest.

## Publisher's note

All claims expressed in this article are solely those of the authors and do not necessarily represent those of their affiliated organizations, or those of the publisher, the editors and the reviewers. Any product that may be evaluated in this article, or claim that may be made by its manufacturer, is not guaranteed or endorsed by the publisher.

## References

[1] Zamorano JL Lancellotti P Rodriguez MD Aboyans V Asteggiano R Galderisi M. 2016 ESC position paper on cancer treatments and cardiovascular toxicity developed under the auspices of the ESC committee for practice guidelines: the task force for cancer treatments and cardiovascular toxicity of the European society of cardiology (ESC). Eur Heart J. (2016) 37:2768–801. 10.1093/eurheartj/ehw211 27567406

[2] Desai MY Windecker S Lancellotti P Bax JJ Griffin BP Cahlon O. Prevention, diagnosis, and management of radiation-associated cardiac disease: JACC scientific expert panel. J Am Coll Cardiol. (2019) 74:905–27. 10.1016/j.jacc.2019.07.006 31416535

[3] Darby SC Ewertz M McGale P Bennet AM Blom-Goldman U Bronnum D. Risk of ischemic heart disease in women after radiotherapy for breast cancer. N Engl J Med. (2013) 368:987–98. 10.1056/NEJMoa1209825 23484825

[4] Venkatesulu BP Mahadevan LS Aliru ML Yang X Bodd MH Singh PK. Radiation-Induced endothelial vascular injury: a review of possible mechanisms. JACC Basic Transl Sci. (2018) 3:563–72. 10.1016/j.jacbts.2018.01.014 30175280 PMC6115704

[5] Sardar P Kundu A Chatterjee S Nohria A Nairooz R Bangalore S. Long-term cardiovascular mortality after radiotherapy for breast cancer: a systematic review and meta-analysis. Clin Cardiol. (2017) 40:73–81. 10.1002/clc.22631 28244595 PMC6490535

[6] van Nimwegen FA Ntentas G Darby SC Schaapveld M Hauptmann M Lugtenburg PJ. Risk of heart failure in survivors of hodgkin lymphoma: effects of cardiac exposure to radiation and anthracyclines. Blood. (2017) 129:2257–65. 10.1182/blood-2016-09-740332 28143884 PMC5418626

[7] Maraldo MV Brodin NP Aznar MC Vogelius IR Munck ARP Petersen PM. Estimated risk of cardiovascular disease and secondary cancers with modern highly conformal radiotherapy for early-stage mediastinal hodgkin lymphoma. Ann Oncol. (2013) 24:2113–18. 10.1093/annonc/mdt156 23619032

[8] Siegel RL Miller KD Jemal A. Cancer statistics, 2020. CA Cancer J Clin. (2020) 70:7–30. 10.3322/caac.21590 31912902

[9] Lally BE Detterbeck FC Geiger AM Thomas CJ Machtay M Miller AA. The risk of death from heart disease in patients with nonsmall cell lung cancer who receive postoperative radiotherapy: analysis of the surveillance, epidemiology, and end results database. Cancer-Am Cancer Soc. (2007) 110:911–7. 10.1002/cncr.22845 17620279

[10] Bradley JD Paulus R Komaki R Masters G Blumenschein G Schild S. Standard-dose versus high-dose conformal radiotherapy with concurrent and consolidation carboplatin plus paclitaxel with or without cetuximab for patients with stage IIIA or IIIB non-small-cell lung cancer (RTOG 0617): a randomised, two-by-two factorial phase 3 study. Lancet Oncol. (2015) 16:187–99. 10.1016/S1470-2045(14)71207-0 25601342 PMC4419359

[11] Dess RT Sun Y Matuszak MM Sun G Soni PD Bazzi L. Cardiac events after radiation therapy: combined analysis of prospective multicenter trials for locally advanced Non-Small-Cell lung cancer. J Clin Oncol. (2017) 35:1395–402. 10.1200/JCO.2016.71.6142 28301264 PMC5455464

[12] Wang K Eblan MJ Deal AM Lipner M Zagar TM Wang Y. Cardiac toxicity after radiotherapy for stage III non-small-cell lung cancer: pooled analysis of dose-escalation trials delivering 70 to 90 Gy. J Clin Oncol. (2017) 35:1387–94. 10.1200/JCO.2016.70.0229 28113017 PMC5455462

[13] Haque W Verma V Fakhreddine M Butler EB Teh BS Simone CN. Trends in cardiac mortality in patients with locally advanced non-small cell lung cancer. Int J Radiat Oncol Biol Phys. (2018) 100:470–7. 10.1016/j.ijrobp.2017.10.031 29353659

[14] Atkins KM Rawal B Chaunzwa TL Lamba N Bitterman DS Williams CL. Cardiac radiation dose, cardiac disease, and mortality in patients with lung cancer. J Am Coll Cardiol. (2019) 73:2976–87. 10.1016/j.jacc.2019.03.500 31196455

[15] Pignon JP Arriagada R Ihde DC Johnson DH Perry MC Souhami RL. A meta-analysis of thoracic radiotherapy for small-cell lung cancer. N Engl J Med. (1992) 327:1618–24. 10.1056/NEJM199212033272302 1331787

[16] Faivre-Finn C Snee M Ashcroft L Appel W Barlesi F Bhatnagar A. Concurrent once-daily versus twice-daily chemoradiotherapy in patients with limited-stage small-cell lung cancer (CONVERT): An open-label, phase 3, randomised, superiority trial. Lancet Oncol. (2017) 18:1116–25. 10.1016/S1470-2045(17)30318-2 28642008 PMC5555437

[17] Yang S Zhang Z Wang Q. Emerging therapies for small cell lung cancer. J Hematol Oncol. (2019) 12:47. 10.1186/s13045-019-0736-3 31046803 PMC6498593

[18] Li C Liu J Lin J Li Z Shang X Wang H. Poor survival of non-small-cell lung cancer patients with main bronchus tumor: a large population-based study. Future Oncol. (2019) 15:2819–27. 10.2217/fon-2019-0098 31393163

[19] Yang L Wang S Gerber DE Zhou Y Xu F Liu J. Main bronchus location is a predictor for metastasis and prognosis in lung adenocarcinoma: a large cohort analysis. Lung Cancer. (2018) 120:22–6. 10.1016/j.lungcan.2018.03.011 29748011 PMC7678407

[20] Sun W Yang X Liu Y Yuan Y Lin D. Primary tumor location is a useful predictor for lymph node metastasis and prognosis in lung adenocarcinoma. Clin Lung Cancer. (2017) 18:e49–55. 10.1016/j.cllc.2016.06.002 27426975

[21] Jan N Guy C Reshko LB Hugo GD Weiss E. Lung and heart dose variability during radiation therapy of non-small cell lung cancer. Int J Radiat Oncol Biol Phys. (2017) 98:683–90. 10.1016/j.ijrobp.2017.02.227 28581410 PMC5527748

[22] Hancock SL Tucker MA Hoppe RT. Factors affecting late mortality from heart disease after treatment of Hodgkin's disease. JAMA. (1993) 270:1949–55. 10.1001/jama.270.16.1949 8411552

[23] Wolbers M Koller MT Stel VS Schaer B Jager KJ Leffondre K. Competing risks analyses: objectives and approaches. Eur Heart J. (2014) 35:2936–41. 10.1093/eurheartj/ehu131 24711436 PMC4223609

[24] Deb S Austin PC Tu JV Ko DT Mazer CD Kiss A. A review of propensity-score methods and their use in cardiovascular research. Can J Cardiol. (2016) 32:259–65. 10.1016/j.cjca.2015.05.015 26315351

[25] Austin PC. The use of propensity score methods with survival or time-to-event outcomes: Reporting measures of effect similar to those used in randomized experiments. Stat Med. (2014) 33:1242–58. 10.1002/sim.5984 24122911 PMC4285179

[26] Ferris MJ Jiang R Behera M Ramalingam SS Curran WJ Higgins KA. Radiation therapy is associated with an increased incidence of cardiac events in patients with small cell lung cancer. Int J Radiat Oncol Biol Phys. (2018) 102:383–90. 10.1016/j.ijrobp.2018.05.066 30191870 PMC6242711

[27] Verma V Fakhreddine MH Haque W Butler EB Teh BS Simone CN. Cardiac mortality in limited-stage small cell lung cancer. Radiother Oncol. (2018) 128:492–7. 10.1016/j.radonc.2018.06.011 29934110

[28] Daitoku K Fukui K Ichinoseki I Munakata M Takahashi S Fukuda I. Radiotherapy-induced aortic valve disease associated with porcelain aorta. Jpn J Thorac Cardiovasc Surg. (2004) 52:349–52. 10.1007/s11748-004-0069-0 15296033

[29] Brosius FR Waller BF Roberts WC. Radiation heart disease. analysis of 16 young (aged 15 to 33 years) necropsy patients who received over 3,500 rads to the heart. Am J Med. (1981) 70:519–30. 10.1016/0002-9343(81)90574-X 6782873

[30] Coblentz C Martin L Tuttle R. Calcified ascending aorta after radiation therapy. AJR Am J Roentgenol. (1986) 147:477–8. 10.2214/ajr.147.3.477 3488649

[31] Ledoux B Dupont M Duplaquet F Pirard L Ocak S Wanet M. Illustration of a fatal radiation-induced lung aneurysm: is central lung stereotactic radiotherapy to be banned? Cancer Radiother. (2019) 23:926–9. 10.1016/j.canrad.2019.05.016 31611052

[32] Kraus RD Hamilton AS Carlos M Ballas LK. Using hospital medical record data to assess the accuracy of the SEER Los Angeles cancer surveillance program for initial treatment of prostate cancer: a small pilot study. Cancer Causes Control. (2018) 29:815–21. 10.1007/s10552-018-1057-5 30022335 PMC7439650

