# Supplementary material for: Primary site as a novel prognostic factor for cardiovascular mortality post-radiotherapy in limited-stage small cell lung cancer: A large population-based study
Source: Front Cardiovasc Med. 2022 Aug 12;9:922811. doi: 10.3389/fcvm.2022.922811 (PMC9411942; doi:10.3389/fcvm.2022.922811)
Supplement: Supplementary file 1 [file Table_1.DOCX]

**Supplementary Table 1: Univariate Cox proportional hazards and Fine-Gray competing risk regression models for predictors of NCVM before and after PSM.**

| **Variables** | **Group** | **Before PSM** | | | | |  | **After PSM** | | | | |
| --- | --- | --- | --- | --- | --- | --- | --- | --- | --- | --- | --- | --- |
|  |  | **Cox proportional hazards (Univariate)** | |  | **Fine-Gray competing risk (Univariate)** | |  | **Cox proportional hazards (Univariate)** | |  | **Fine-Gray competing risk (Univariate)** | |
|  |  | **HR (95% CI)** | ***P* value** |  | **HR (95% CI)** | ***P* value** |  | **HR (95% CI)** | ***P* value** |  | **HR (95% CI)** | ***P* value** |
| Primary site | UL/ML | Reference |  |  | Reference |  |  | Reference |  |  | Reference |  |
|  | MB/LL | 1.04 (0.98-1.11) | 0.189 |  | 1.03 (0.96-1.09) | 0.442 |  | 1.06 (0.98-1.14) | 0.137 |  | 1.04 (0.97-1.11) | 0.324 |
| Age years | ≤ 57 | Reference |  |  | Reference |  |  | Reference |  |  | Reference |  |
|  | > 57 | 1.18 (1.11-1.26) | < 0.001 |  | 1.17 (1.10-1.24) | < 0.001 |  | 1.18 (1.09-1.26) | < 0.001 |  | 1.15 (1.07-1.24) | < 0.001 |
| Sex | Male | Reference |  |  | Reference |  |  | Reference |  |  | Reference |  |
|  | Female | 0.82 (0.77-0.87) | < 0.001 |  | 0.84 (0.79-0.89) | < 0.001 |  | 0.81 (0.76-0.87) | < 0.001 |  | 0.83 (0.77-0.89) | < 0.001 |
| Race | White | Reference |  |  | Reference |  |  | Reference |  |  | Reference |  |
|  | Black | 1.06 (0.96-1.17) | 0.229 |  | 1.04 (0.95-1.15) | 0.374 |  | 1.08 (0.96-1.21) | 0.209 |  | 1.05 (0.93-1.18) | 0.445 |
|  | Other | 0.96 (0.83-1.12) | 0.607 |  | 0.98 (0.86-1.12) | 0.773 |  | 0.94 (0.79-1.12) | 0.495 |  | 0.95 (0.81-1.12) | 0.573 |
| Marriage | Unmarried | Reference |  |  | Reference |  |  | Reference |  |  | Reference |  |
|  | Married | 0.93 (0.87-0.99) | 0.025 |  | 0.94 (0.88-1.00) | 0.042 |  | 0.91 (0.85-0.99) | 0.019 |  | 0.92 (0.86-0.99) | 0.031 |
|  | Unknown | 0.95 (0.79-1.14) | 0.593 |  | 0.95 (0.80-1.14) | 0.610 |  | 0.93 (0.75-1.16) | 0.538 |  | 0.93 (0.75-1.16) | 0.529 |
| Year of diagnosis | 1988-2003 | Reference |  |  | Reference |  |  | Reference |  |  | Reference |  |
|  | 2004-2013 | 0.80 (0.75-0.85) | < 0.001 |  | 0.81 (0.76-0.85) | < 0.001 |  | 0.82 (0.76-0.88) | < 0.001 |  | 0.82 (0.77-0.89) | < 0.001 |
| AJCC stage | I-II | Reference |  |  | Reference |  |  | Reference |  |  | Reference |  |
|  | III | 1.29 (1.18-1.41) | < 0.001 |  | 1.29 (1.18-1.40) | < 0.001 |  | 1.30 (1.17-1.44) | < 0.001 |  | 1.30 (1.18-1.44) | < 0.001 |
| Laterality | Left | Reference |  |  | Reference |  |  | Reference |  |  | Reference |  |
|  | Right | 1.01 (0.95-1.07) | 0.821 |  | 1.01 (0.95-1.08) | 0.696 |  | 0.98 (0.91-1.06) | 0.629 |  | 0.99 (0.92-1.06) | 0.679 |

NCVM: cardiovascular mortality; PSM: propensity score matching; HR: hazard ratio; CI: confidence interval; AJCC: American Joint Committee on Cancer; UL/ML: upper lobe/middle lobe; MB/LL: main bronchus/lower lobe.

**Supplementary Table 2: Multivariate Cox proportional hazards and Fine-Gray competing risk regression models for predictors of NCVM before and after PSM.**

| **Variables** | **Group** | **Before PSM** | | | | |  | **After PSM** | | | | |
| --- | --- | --- | --- | --- | --- | --- | --- | --- | --- | --- | --- | --- |
|  |  | **Cox proportional hazards (Multivariate)** | |  | **Fine-Gray competing risk (Multivariate)** | |  | **Cox proportional hazards (Multivariate)** | |  | **Fine-Gray competing risk (Multivariate)** | |
|  |  | **HR (95% CI)** | ***P* value** |  | **HR (95% CI)** | ***P* value** |  | **HR (95% CI)** | ***P* value** |  | **HR (95% CI)** | ***P* value** |
| Primary site | UL/ML | Reference |  |  | Reference |  |  | Reference |  |  | Reference |  |
|  | MB/LL | 1.05 (0.99-1.12) | 0.127 |  | 1.03 (0.97-1.10) | 0.358 |  | 1.07 (1.00-1.15) | 0.063 |  | 1.05 (0.98-1.13) | 0.184 |
| Age years | ≤ 57 | Reference |  |  | Reference |  |  | Reference |  |  | Reference |  |
|  | > 57 | 1.18 (1.11-1.26) | < 0.001 |  | 1.16 (1.10-1.24) | < 0.001 |  | 1.18 (1.10-1.27) | < 0.001 |  | 1.15 (1.07-1.24) | < 0.001 |
| Sex | Male | Reference |  |  | Reference |  |  | Reference |  |  | Reference |  |
|  | Female | 0.82 (0.77-0.87) | < 0.001 |  | 0.83 (0.78-0.89) | < 0.001 |  | 0.80 (0.75-0.87) | < 0.001 |  | 0.83 (0.77-0.89) | < 0.001 |
| Race | White | Reference |  |  | Reference |  |  | Reference |  |  | Reference |  |
|  | Black | 1.04 (0.94-1.15) | 0.432 |  | 1.02 (0.93-1.13) | 0.641 |  | 1.06 (0.94-1.19) | 0.370 |  | 1.02 (0.91-1.15) | 0.711 |
|  | Other | 0.92 (0.79-1.07) | 0.277 |  | 0.95 (0.83-1.08) | 0.413 |  | 0.90 (0.75-1.07) | 0.237 |  | 0.92 (0.78-1.08) | 0.320 |
| Marriage | Unmarried | Reference |  |  | Reference |  |  | Reference |  |  | Reference |  |
|  | Married | 0.89 (0.84-0.95) | < 0.001 |  | 0.90 (0.84-0.96) | 0.001 |  | 0.88 (0.81-0.95) | 0.001 |  | 0.89 (0.82-0.96) | 0.002 |
|  | Unknown | 0.93 (0.78-1.12) | 0.460 |  | 0.94 (0.79-1.12) | 0.491 |  | 0.91 (0.73-1.14) | 0.407 |  | 0.91 (0.74-1.13) | 0.406 |
| Year of diagnosis | 1988-2003 | Reference |  |  | Reference |  |  | Reference |  |  | Reference |  |
|  | 2004-2013 | 0.79 (0.75-0.84) | < 0.001 |  | 0.80 (0.75-0.85) | < 0.001 |  | 0.81 (0.75-0.88) | < 0.001 |  | 0.82 (0.76-0.88) | < 0.001 |
| AJCC stage | I-II | Reference |  |  | Reference |  |  | Reference |  |  | Reference |  |
|  | III | 1.33 (1.22-1.45) | < 0.001 |  | 1.32 (1.21-1.44) | < 0.001 |  | 1.33 (1.20-1.48) | < 0.001 |  | 1.33 (1.20-1.48) | < 0.001 |
| Laterality | Left | Reference |  |  | Reference |  |  | Reference |  |  | Reference |  |
|  | Right | 0.99 (0.93-1.06) | 0.860 |  | 1.00 (0.94-1.06) | 0.995 |  | 0.97 (0.90-1.04) | 0.427 |  | 0.97 (0.91-1.05) | 0.470 |

NCVM: cardiovascular mortality; PSM: propensity score matching; HR: hazard ratio; CI: confidence interval; AJCC: American Joint Committee on Cancer; UL/ML: upper lobe/middle lobe; MB/LL: main bronchus/lower lobe.
